# Involvement of muscarinic receptors in psychomotor hyperactivity in dopamine-deficient mice

**DOI:** 10.1186/s13041-022-00984-x

**Published:** 2022-11-29

**Authors:** Masayo Fujita, Yukiko Ochiai, Yoko Hagino, Kazuto Kobayashi, Geoffrey Pavey, Brian Dean, Kazutaka Ikeda

**Affiliations:** 1 Addictive Substance Project, Tokyo Metropolitan Institute of Medical Science, 2-1-6 Kamikitazawa, Setagaya-Ku, Tokyo, 156-8506 Japan; 2 Department of Neurology, Tokyo Metropolitan Neurological Hospital, Tokyo, Japan; 3 Department of Molecular Genetics, Institute of Biomedical Sciences, Fukushima Medical University, Fukushima, Japan; 4 Florey Institute of Neuroscience and Mental Health, Parkville, Australia

**Keywords:** Dopamine deficient, Muscarinic signaling, Muscarinic receptor, Binding assay, Hyperactivity

## Abstract

Dopamine-deficient (DD) mice exhibit psychomotor hyperactivity that might be related to a decrease in muscarinic signaling. In the present study, muscarinic acetylcholine receptor M2 (CHRM2) density decreased in the cortex in DD mice. This is significant because cortical CHRM2 acts as an autoreceptor; therefore, changes in CHRM2 levels could alter acetylcholine in DD mice. We also found that the CHRM1/CHRM4 agonist xanomeline and CHRM2 agonist arecaidine propargyl ester tosylate inhibited hyperactivity in DD mice, suggesting that postsynaptic CHRM1 and CHRM2 and presynaptic CHRM2 may be involved in hyperactivity in DD mice.

**Supplementary Information:**

The online version contains supplementary material available at 10.1186/s13041-022-00984-x.

## Background

A decrease in dopamine levels is generally considered to impair motor function. We used a dopamine-deficient (DD) mouse model in which the tyrosine hydroxylase (TH) gene is knocked out but TH expression is rescued in noradrenergic and adrenergic neurons by introducing a transgene that expresses TH under the dopamine β-hydroxylase promotor [1]. Using this model, we previously reported that DD mice, which have extremely low levels of dopamine in the brain, are hyperactive when placed in a novel environment [2]. Hyperactivity in DD mice was not suppressed by typical antipsychotics but was reduced by clozapine, suggesting that this psychomotor hyperactivity might reflect a treatment-resistant schizophrenia-like phenotype [2].

Unlike typical antipsychotic drugs, clozapine and its metabolites target muscarinic acetylcholine receptors (CHRMs), which mediate the regulation of ion channels by activating signal-transducing G proteins and intracellular effector systems [3, 4]. Thus, clozapine and/or its metabolites could exert therapeutic effects by exerting actions on CHRMs, which are suggested to be involved in the pathophysiology of schizophrenia [5]. We previously reported that hyperactivity in DD mice in a novel environment was inhibited by oxotremorine [2], a nonselective CHRM agonist [6], and acetylcholine levels decreased in DD mice [2]. Acetylcholine plays a key role in various nervous system functions, including the contraction of skeletal muscles, emotion, perception, cognition, learning, and memory. These results suggest that a decrease in CHRM activation that is caused by a decrease in acetylcholine could be involved in hyperactivity in DD mice. The present study investigated whether CHRM density is disturbed and whether a CHRM subtype-selective agonist or antagonist affects locomotor activity in DD mice.

## Methods

Male and female DD mice on a C57BL/6J background were maintained on daily intraperitoneal injections of 50 mg/kg l-3,4-dihydroxyphenylalanine (l-DOPA; Nacalai Tesque, Kyoto, Japan) until 6 weeks of age. The DD mice were then given a paste diet that was soaked in water and contained 500 mg l-DOPA, 125 mg benserazide (Fujifilm Wako Pure Chemical, Tokyo, Japan), and 250 mg ascorbic acid (Nakalai Tesque) in 1 kg of powdered feed. The DD mice were given a 50 mg/kg l-DOPA injection 72 h before testing. For the binding assays, brain samples were collected 72 h after the l-DOPA injection and then stored at − 80 °C until use. Levels of [^3^H]pirenzepine (CHRM1 antagonist; DuPont, Melbourne, Australia), [^3^H]AFDX-384 (CHRM2 antagonist; DuPont), and [^3^H]4-DAMP (CHRM3 antagonist; DuPont) binding were measured using established methodologies [7]. Locomotor activity was measured in a novel environment as described previously [2]. In the present study, we used a commercially available CHRM subtype-selective agonist and antagonist. Xanomeline (CHRM1/CHRM4 agonist; 10 mg/kg; Tocris Bioscience, Bristol, UK), arecaidine propargyl ester tosylate (CHRM2 agonist; 5 mg/kg; Tocris Bioscience), VU0255035 (CHRM1 antagonist; 10 mg/kg; Tocris Bioscience), and AQRA-741 (CHRM2 antagonist; 1 mg/kg; Tocris Bioscience) were dissolved in saline and administered subcutaneously. The dose of each drug was determined according to doses that were used in mice in previous studies or doses that decreased locomotor activity in wildtype (WT) mice [8–10]. The statistical analyses were performed using Student’s *t*-test or two-way repeated-measures analysis of variance (ANOVA) followed by the Scheffe post hoc test. Values of *p* < 0.05 were considered statistically significant. The data were analyzed using BellCurve for Excel software (Social Survey Research Information, Tokyo, Japan).

## Results and discussion

**Fig. 1:**
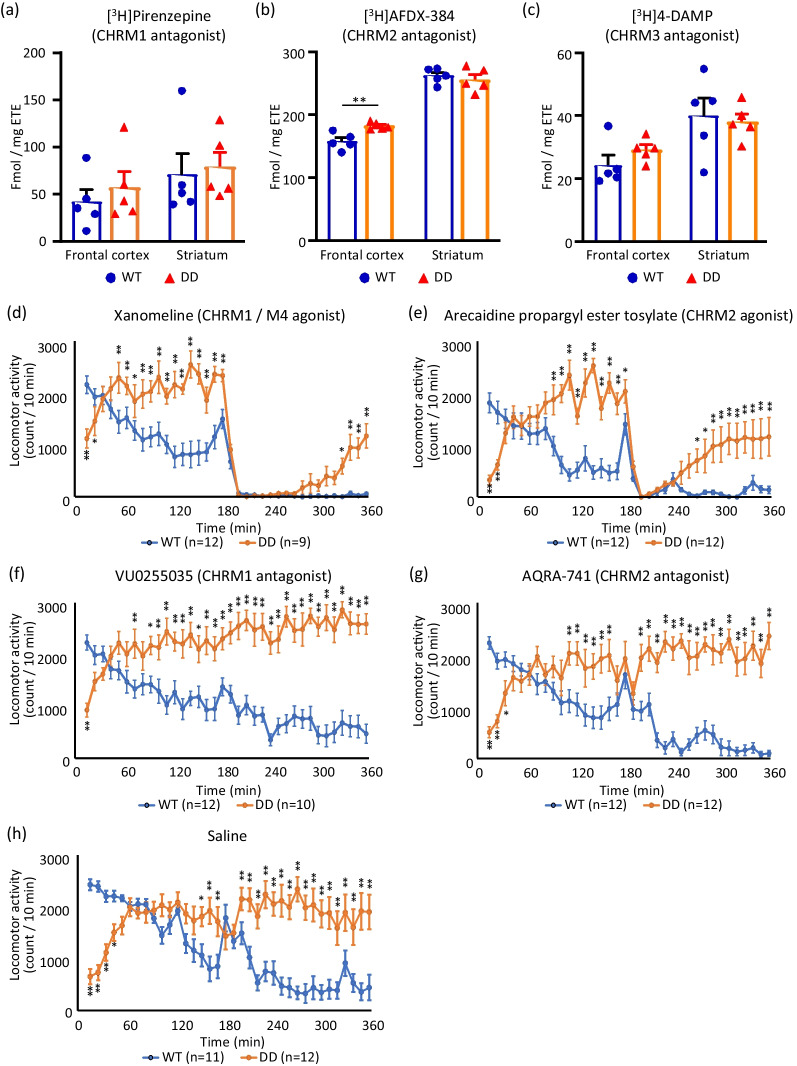
Effects of CHRM subtype-selective agonists and antagonists on CHRM density and locomotor activity in DD mice. (**a**–**c**) Binding assays with [^3^H]pirenzepine, [^3^H]AFDX-384, and [^3^H]4-DAMP were conducted. WT mice: *n* = 5, DD mice: *n* = 5. **p < 0.01 (Student’s *t*-test). The data are expressed as the mean + SEM with data point overlap. (**d**-**h**) Change in locomotor activity in WT mice (*n* = 11–12) and DD mice (*n* = 9–12) following xanomeline, arecaidine propargyl ester tosylate, VU0255035, AQRA-741, and saline treatment. *p < 0.05, **p < 0.01 (two-way repeated-measures ANOVA). The data are expressed as the mean ± SEM

No significant differences in [^3^H]pirenzepine or [^3^H]4-DAMP binding were found in the cortex or striatum between DD and WT mice. The binding of [^3^H]AFDX-384 was significantly higher in the cortex but not striatum in DD mice (Fig. 1a–c). These data suggest that CHRM2 levels in the cortex increased in DD mice, whereas CHRM1, CHRM3, and CHRM4 levels were unaltered in the cortex and striatum in DD mice. Notably, our previous data from CHRM knockout mice showed that the methodology we used in the present study means that [^3^H]AFDX-384 preferentially binds to CHRM2 [11, 12]. Therefore, our data suggest that CHRM2 levels are higher in the cortex in DD mice (*p* = 0.0048). The cerebral cortex receives cholinergic afferents from the nucleus of Meynert. This is important because CHRM2 in the cortex predominantly acts as a cholinergic autoreceptor [13] that aids in the regulation of acetylcholine from presynaptic neurons. Higher levels of CHRM2 in DD mice could reflect a compensatory increase in sensitivity of the autoreceptor-driven feedback loop in an attempt to reduce acetylcholine levels in DD mice.

Hyperactivity in DD mice was reduced by treatment with xanomeline (Fig. 1d) and arecaidine propargyl ester tosylate (Fig. 1e). However, the effect of arecaidine propargyl ester tosylate was shorter than xanomeline. Hyperactivity was not reduced by treatment with VU0255035 (Fig. 1f) or AQRA-741 (Fig. 1g). Xanomeline is a CHRM1/CHRM4 agonist, arecaidine propargyl ester tosylate is a CHRM2 agonist, VU0255035 is a CHRM1 antagonist, and AQRA-741 is a CHRM2 antagonist. Based on these data, low levels of acetylcholine in DD mice may cause a maximal change in locomotion that is not further influenced by a receptor antagonist that lowers cholinergic activity in the brain. In contrast, the reversal of hyperactivity by a CHRM2 agonist and CHRM1/CHRM4 agonist suggests that CHRM2, CHRM1, and/or CHRM4 are involved in mediating hyperactivity in DD mice. Saline treatment alone did not affect hyperactivity in DD mice (Fig. 1h). All raw data are included in Additional file 1.

Xanomeline effectively suppressed hyperactivity in DD mice. Xanomeline treatment alone [14] and combined with a peripheral CHRM antagonist [15] effectively reduced clinical symptoms of schizophrenia in humans. Therefore, our preliminary data suggest that DD mice may be a valid model for studying the mechanisms by which CHRM agonists exert therapeutic effects in schizophrenia patients.

In conclusion, CHRM2 density increased in DD mice, possibly reflecting a physiological response to low levels of acetylcholine. Our data suggest that DD mice may be a useful model for studying cholinergic abnormalities that have been reported to exist in the central nervous system in schizophrenia patients.

## Supplementary Information

**Additional file 1.** All raw data are included in Additional file 1

## Data Availability

All data generated or analyzed during this study are included in this published article and its Additional file.

## Abbreviations

DD: Dopamine deficient
TH: Tyrosine hydroxylase
CHRM: Muscarinic acetylcholine receptor
l-DOPA: l-3,4-Dihydroxyphenylalanine
WT: Wildtype
